# The Mesothelioma Systemic Inflammation Score Is Independently Associated with Overall Survival and Predicts Benefit of Multimodality Treatment in Pleural Mesothelioma

**DOI:** 10.3390/cancers17081371

**Published:** 2025-04-20

**Authors:** Berta Mosleh, Katharina Sinn, Anna Cho, Anton Reiner, Ariane Steindl, Christian Lang, Sabine Zöchbauer-Müller, Karin Dieckmann, Joachim Widder, Helmut Prosch, Balazs Dome, Karin Schelch, Clemens Aigner, Thomas Klikovits, Michal Benej, Stefan Watzka, Martin Filipits, Servet Bölükbas, Pavla Sarova, Daniela Gompelmann, Michael Grusch, Mir Alireza Hoda

**Affiliations:** 1 Department of Thoracic Surgery, Comprehensive Cancer Center Vienna, Medical University of Vienna, 1090 Vienna, Austria; katharina.sinn@meduniwien.ac.at (K.S.); balazs.dome@meduniwien.ac.at (B.D.); karin.schelch@meduniwien.ac.at (K.S.); clemens.aigner@meduniwien.ac.at (C.A.); thomas.klikovits@meduniwien.ac.at (T.K.); mir.hoda@meduniwien.ac.at (M.A.H.); 2 Department of Neurosurgery, Medical University of Vienna, 1090 Vienna, Austria; anna.cho@meduniwien.ac.at; 3 Division of Oncology, Department of Internal Medicine I, Comprehensive Cancer Center Vienna, Medical University of Vienna, 1090 Vienna, Austria; ariane.steindl@meduniwien.ac.at (A.S.); sabine.zoechbauer-mueller@meduniwien.ac.at (S.Z.-M.); 4 Division of Pulmonology, Department of Internal Medicine II, Comprehensive Cancer Center Vienna, Medical University of Vienna, 1090 Vienna, Austria; christian.lang@meduniwien.ac.at (C.L.); pavla.sarova@meduniwien.ac.at (P.S.); daniela.gompelmann@meduniwien.ac.at (D.G.); 5 Department of Radiation Oncology, Comprehensive Cancer Center Vienna, Medical University of Vienna, 1090 Vienna, Austria; karin.dieckmann@meduniwien.ac.at (K.D.); joachim.widder@meduniwien.ac.at (J.W.); 6 Department of Biomedical Imaging and Image-Guided Therapy, Comprehensive Cancer Center Vienna, Medical University of Vienna, 1090 Vienna, Austria; helmut.prosch@meduniwien.ac.at; 7 National Koranyi Institute of Pulmonology, 1121 Budapest, Hungary; 8 Department of Thoracic Surgery, National Institute of Oncology-Semmelweis University, 1122 Budapest, Hungary; 9 Center for Cancer Research, Medical University of Vienna, 1090 Vienna, Austria; martin.filipits@meduniwien.ac.at (M.F.); michael.grusch@meduniwien.ac.at (M.G.); 10 Department of Thoracic Surgery, Karl Landsteiner Institute for Clinical and Translational Thoracic Surgery Research, Clinic Floridsdorf, 1210 Vienna, Austria; michal.benej@gesundheitsverbund.at (M.B.); stefan.watzka@gesundheitsverbund.at (S.W.); 11 Department of Thoracic Surgery, University Medical Center Essen-Ruhrlandklinik, University Duisburg-Essen, 45239 Essen, Germany; servet.boeluekbas@rlk.uk-essen.de

**Keywords:** malignant pleural mesothelioma, biomarkers, systemic inflammation score, prognostic score, predictive score

## Abstract

Background/Objectives: Malignant pleural mesothelioma (MPM) remains challenging to treat, with a poor prognosis. As controversy about clinical management continues, predictive biomarkers for patient selection to indicate the benefit of treatment modalities are urgently needed. Methods: In a retrospective analysis of 195 patients between 1994 and 2020 at the Department of Thoracic Surgery, Medical University of Vienna, Austria, the Mesothelioma Systemic Inflammation Score (MSIS)—consisting of pretreatment neutrophil-to-lymphocyte ratio (NLR), platelet-to-lymphocyte ratio (PLR), C-reactive protein (CRP), and fibrinogen—was tested for its prognostic and predictive significance. The prognostic impact of MSIS was subsequently validated in an independent cohort of 80 patients treated at the Department of Thoracic Surgery, Karl Landsteiner Institute for Clinical and Translational Thoracic Surgery Research, Clinic Floridsdorf, Vienna, Austria. Results: Median overall survival (OS) was 14 months for the entire cohort (95% CI: 11.4–16.6). Patients undergoing multimodality treatment including macroscopic complete resection had a longer OS (22.3 months, 95% CI: 18.6–26.0; *p* < 0.001). In multivariable analysis, MSIS (*p* < 0.001), disease stage (*p* = 0.001), and the type of treatment (*p* = 0.004) were confirmed as independent predictors for OS. Higher MSIS was associated with shorter OS (*p* < 0.001). Significant survival benefit of multimodality regimens including surgery was limited to patients with low MSIS. Among patients with low (≤ 2) MSIS, multimodality therapy was associated with significantly prolonged OS when compared with chemo- and/or radiotherapy alone (25.8 months [95% CI: 16.4–35.3] vs. 14.4 months [95% CI: 10.4–18.4], *p* < 0.001). In contrast, among patients with elevated MSIS, no survival benefit was achieved by surgery over conservative treatment (11.8 months [95% CI: 8.3–15.3] vs. 8.2 months [95% CI: 5.2–11.3], *p* = 0.233). The ability of MSIS to predict survival was equivalent between the baseline and the independent validation cohort (*p* < 0.001). Conclusions: The Mesothelioma Systemic Inflammation Score was found to be an independent prognostic score in pleural mesothelioma, predicting benefit from macroscopic complete resection as part of multimodality treatment in distinct patients.

## Simple Summary

Few malignancies generate as many controversies about clinical management as pleural mesothelioma. The merits of multimodality treatment protocols have been continuously discussed, often with limited evidence from prospective trials. Given that the first randomized prospective studies investigating the role of surgery in mesothelioma produced debatable results, it may be too soon to retire the concept of multimodality treatment protocols including surgery for selected candidates. Accurate prognostication could improve treatment allocation, offering multimodality therapy pathways or sparing patients from aggressive and potentially morbid treatment strategies. The aim of our study was to validate the prognostic and predictive power of a novel inflammation-based score—the Mesothelioma Systemic Inflammation Score (MSIS)—in a large cohort of pleural mesothelioma patients treated in a high-volume institution. The prognostic impact of MSIS was further validated in an independent cohort. The pretreatment Mesothelioma Systemic Inflammation Score was found to predict treatment benefits of surgery within multimodality treatment by stratifying patient groups with significant survival differences.

## 1. Introduction

Malignant pleural mesothelioma (MPM) is a highly aggressive, often asbestos-induced malignancy with dismal outcomes. The incidence may still rise in some countries, reflecting the global trend of commercial asbestos use [1,2]. The prognosis is poor, with a median overall survival time (OS) ranging from 10 to 22 months depending on the stage, histology, sex, and type of treatment [3,4]. Identifying clinically useful biomarkers and prognostic scores for this disease is imperative to facilitate early diagnosis and guide treatment allocation. Prognostic factors such as age, sex, histologic subtype, tumor stage, performance status, and several routine laboratory parameters have been validated in different population studies [5,6,7,8,9]. Also, systemic inflammatory response has been shown to have a pivotal role in cancer outcome. In consequence, inflammatory scores have been proposed to indicate prognosis [5,6,7,10,11,12,13]. Prognostic scoring systems such as the European Organization for Research and Treatment of Cancer (EORTC) and the Cancer and Leukemia Group B (CALGB) have been designed to assess clinical outcomes in cancer patients. Prognostic factors for pleural mesothelioma included in these models are performance status, age, sex, histologic subtype, symptoms, and blood parameters, particularly LDH, platelet, and white blood cell counts. The practical use of these scoring systems is limited in daily clinical management [14,15]. Furthermore, the literature lacks large-scale studies evaluating the prognostic value of systemic inflammation scores in MPM. A lack of homogeneity in the optimal cut-off values also exists. 

Inflammation scores, namely the neutrophil-to-lymphocyte ratio (NLR), the platelet-to-lymphocyte ratio (PLR), the lymphocyte-to-monocyte ratio (LMR), and the modified Glasgow prognostic score (mGPS), as well as inflammatory biomarkers including C-reactive protein (CRP), fibrinogen, and albumin, have been investigated as independent prognostic factors [16,17,18,19,20,21,22,23,24,25,26]. In particular, NLR, PLR, and LMR have been studied for their prognostic significance in MPM [17,27,28]. In different malignancies, including lung cancer, prostate cancer, renal cell carcinoma, esophageal and gastric cancer, inflammation-based scores have been suggested as predictive factors of patients´ outcomes [29,30,31,32,33,34,35,36,37,38]. Consequently, establishing rational and easily available prognostic systems with clinical utility is of growing interest.

The aim of this study was to validate the prognostic and predictive power of our novel inflammation-based score, the Mesothelioma Systemic Inflammation Score (MSIS), in a large retrospective cohort of MPM patients treated in a high-volume institution. 

## 2. Materials and Methods

### 2.1. Patients and Methods 

In this retrospective study, patients with histologically confirmed MPM, diagnosed between 1994 and 2020, at the Department of Thoracic Surgery, Medical University of Vienna, Austria, were included. Clinical data were consecutively collected from hospital databases and medical records including age, sex, histologic subtype, ECOG performance status, disease stage, diagnostic methods, treatment approaches, symptoms, tumor site, and laboratory parameters. All patients were (re)staged according to the eighth edition of the pleural mesothelioma tumor, node, metastasis (TNM) staging system developed by the International Mesothelioma Interest Group (IMIG) and the International Association for the Study of Lung Cancer (IASLC) [4,39,40,41]. For staging purposes, computed tomography (CT) of the chest and abdomen as well as magnetic resonance imaging (MRI) of the brain or cranial CT scans before 2015 and positron emission tomography CT scans (PET-CT) and brain MRIs after 2015 were performed. This study was approved by the Ethics Committee of the Medical University of Vienna (EK 2137/2022, approval date 31 March 2024) according to the Declaration of Helsinki. 

All patients were presented at thoracic oncology multidisciplinary tumor boards, including thoracic surgeons, oncologists, radiation oncologists, radiologists, and pulmonologists, to define treatment strategies. Patients were stratified to treatment protocols by a comprehensive assessment of the histologic subtype of the disease, stage, tumor volume, and resectability according to the latest guidelines as well as co-morbidities and performance status. Patients aged between 18 and 75 years. 

Treatment strategies included multimodality treatment, chemotherapy and/or palliative radiotherapy, and best supportive care (BSC). Multimodality treatment consisted of neoadjuvant platinum-based chemotherapy combined with pemetrexed, subsequent macroscopic complete resection, and adjuvant hemithoracic radiation therapy. Extrapleural pneumonectomy was systematically performed following a standard technique defined by en bloc resection of the entire lung, visceral and parietal pleura, pericardium and diaphragm, and subsequent reconstruction of the pericardium and diaphragm with prosthetic patches. Patients with an ECOG performance status of 0–1, adequate pulmonary function (predicted postoperative forced expiratory volume in 1s [FEV1] and a diffusing capacity for carbon monoxide [DLCO] > 45%), normal cardiac function (ejection fraction > 55% and no major heart valve disease), no major organ dysfunctions, and no history of other malignancies were eligible for multimodality treatment. 

BSC was defined as symptom relief and palliative care without active anticancer treatment, including pleural effusion and ascites management, as well as pain management to improve quality of life.

### 2.2. Prognostic Scores 

**Figure 1:**
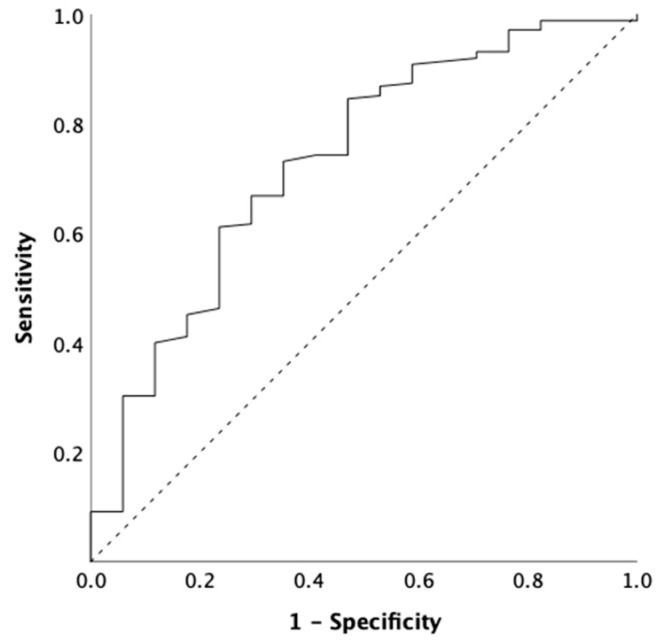
The optimal cut-off value for fibrinogen pretreatment. ROC curve showing the sensitivity and specificity of serum fibrinogen to determine patients with elevated and non-elevated values (AUC: 0.732; 95% CI: 0.60–0.86; *p* < 0.001). Abbreviations: ROC = receiver operating characteristic; AUC: area under the curve; CI = confidence interval.

For all patients, pretreatment laboratory parameters and prognostic scores were evaluated. Laboratory parameters were measured at the date of diagnostic procedures before surgical biopsies and talc pleurodesis to eliminate their potential impact on the inflammatory response. In accordance with the literature, the optimal cut-off value was defined as 1 mg/dL for CRP, 5 for NLR, 160 for PLR, and 2.6 for LMR [17,25,28,42,43,44,45,46,47]. For fibrinogen, the optimal cut-off value was defined as 537.5 mg/dL by the receiver operating characteristic (ROC) curve analysis (Figure 1). Based on these cut-off values validated by Cox regression analysis in our cohort, we established a novel pretreatment inflammatory scoring system, named the Mesothelioma Systemic Inflammation Score (MSIS), consisting of NLR, PLR, CRP, and fibrinogen. Patients with elevated values received one point for each component, resulting in a scoring system of 0–4 in total (Table 1).

### 2.3. Validation Cohort

External validation of the Mesothelioma Systemic Inflammation Score was performed in an independent cohort of patients (*n* = 80) treated at the Department of Thoracic Surgery, Karl Landsteiner Institute for Clinical and Translational Thoracic Surgery Research, Clinic Floridsdorf, Vienna, Austria. Treatment approaches included multimodality treatment (MMT), chemo- and/or radiotherapy (CTH and/or RT), or best supportive care (BSC).

### 2.4. Statistical Analysis

Categorical data were displayed as counts and percentages and continuous variables as median and interquartile ranges (IQRs). Previously published, widely accepted cut-off values were used when available. In case of a lack of substantial evidence from the literature, the optimal cut-off values were determined by the receiver operating characteristic curve (ROC). Fisher´s exact test was performed to assess the relationship between pretreatment MSIS and clinical characteristics of patients. The Cox proportional hazard regression model was used for univariable and multivariable survival analyses to identify independent factors correlated with overall survival (OS). OS was calculated as the time from the definitive histopathological diagnosis to the date of death or last follow-up. Disease-free survival (DFS) was defined from the time of the end of treatment to the time of pathologically or clinically proven recurrence. Survival analyses were displayed as Kaplan–Meier curves. Differences were considered statistically significant for *p*-values < 0.05. All statistical analyses were performed using the SPSS 27.0 software system (SPSS Inc., Chicago, IL, USA). Graphics were generated with GraphPad Prism version 8.4.3. (GraphPad Software, Boston, MA, USA). 

## 3. Results

### 3.1. Patient Characteristics

Details on baseline characteristics are displayed in Table 2. In total, 195 (147 male, 75%) consecutive histologically confirmed MPM patients with sufficient clinical data could be enrolled. The median age was 67 years (IQR 58–74). At the time of diagnosis, 137/195 (70%) patients presented with pleural effusions. Other common symptoms were dyspnea (121/195, 62%) followed by chest pain (76/195, 39%) and cough (36/195, 19%). A reduced ECOG performance status (ECOG PS 1–3) could be observed in 77 patients (40%). 96 patients (49%) were diagnosed with early-stage disease (Stage I–II). The most common diagnostic method was video-assisted thoracoscopy (VATS; 160/195, 82%), followed by the thoracotomy approach (35/195, 18%). The majority of patients presented with an epithelioid subtype (*n* = 144, 74%).

### 3.2. Treatment Strategy

All patients were allocated to either multimodality treatment (MMT), chemo- and/or radiotherapy (CTH and/or RT), or best supportive care (BSC). In total, 90 patients (90/195, 46%) received surgery within a multimodality treatment approach. Extrapleural pneumonectomy (EPP) was the most commonly performed procedure (*n* = 73). Following a shift from lung-sacrificing to lung-sparing surgery, extended pleurectomy/decortication (EPD) and pleurectomy/decortication (P/D) were completed in 12 and 5 patients, respectively. We report six (8.2%) perioperative deaths (within 30 days) following EPP. Lethal adverse events included pulmonary embolism (*n* = 2, 2.7%), acute respiratory distress syndrome (ARDS; *n* = 3, 4.1%), and acute heart failure (*n* = 1, 1.4%). In 80 cases (41%), chemotherapy and/or radiation was proposed; 25 patients (13%) received best supportive care.

### 3.3. Survival and Prognostic Scores

The median OS was 14 months for the entire cohort (95% CI; 11.4–16.6) with a survival rate of 56% at 1 year, 17% at 3 years, and 10% at 5 years, respectively. 

The median OS and DFS of patients undergoing multimodality treatment protocols including macroscopic complete resection were 22.3 months (95% CI; 18.6–26.0) and 15.7 months (95% CI; 12.7–18.7), respectively. 

**Figure 2:**
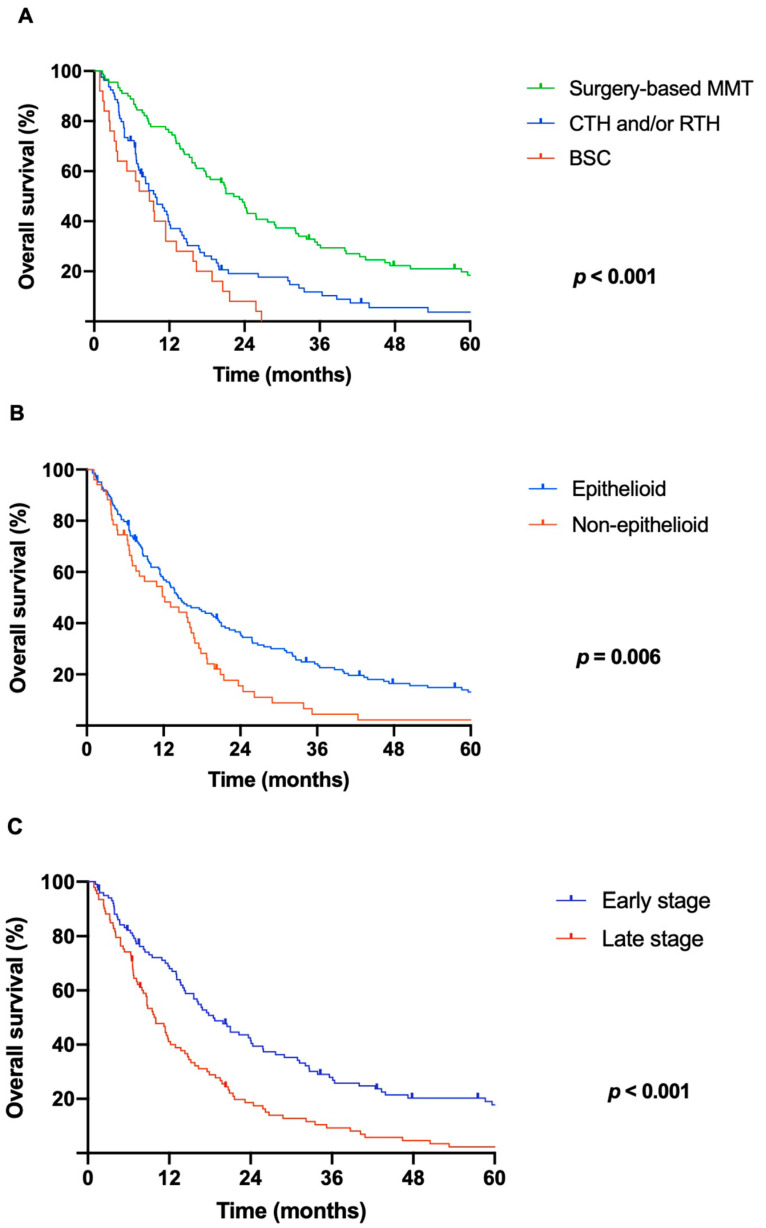
(**A**) Kaplan–Meier survival analysis for patients after different treatment modalities. Patients undergoing multimodality treatment protocols including macroscopic complete resection survived significantly longer (median OS: 22.3 months; 95% CI: 18.6–26.0; *p* < 0.001) when compared to patients treated with chemo- and/or radiotherapy alone (9.8 months, 95% CI: 6.7–12.8) or those receiving best supportive care (8.8 months, 95% CI: 4.2–13.5). (**B**) Kaplan–Meier survival analysis showed significant overall survival benefit in patients with epithelioid histology compared to patients with non-epithelioid subtypes (14.3 months [95% CI: 10.5–18.1] vs. 12.2 months [95% CI: 6.1–18.3]; *p* = 0.006). (**C**) Kaplan–Meier survival analysis for patients with early- vs. late-stage disease: median survival time of 21 months [95% CI: 15.3–26.7] vs. 9.5 months [95% CI: 7.0–12.9]; *p* < 0.001. Abbreviations: MMT = multimodality treatment; CTH = chemotherapy; RTH = radiotherapy, BSC = best supportive care; OS = overall survival; CI = confidence interval.

Notably, patients after multimodality therapy including surgery survived significantly longer (*p* < 0.001, median OS 22.3 months) compared to patients treated with chemo- and/or radiotherapy alone (9.8 months) or those receiving best supportive care (8.8 months; Figure 2A).

Significant overall survival benefit was observed in patients with epithelioid histology compared to patients with non-epithelioid subtypes (14.3 months vs. 12.2 months, *p* = 0.006; Figure 2B) as well as in early (I–II) tumor stages compared to advanced (III–IV) stages (21 months vs. 9.5 months, *p* < 0.001; Figure 2C). 

Elevated fibrinogen (≥537.5 mg/dL; 10 vs. 20.5 months, *p* < 0.001), CRP (≥1 mg/dL; 11.9 vs. 20.5 months, *p* = 0.004), NLR (≥5; 11.4 vs. 16.1 months, *p* = 0.006), and PLR (≥160; 12.9 vs. 20.2 months: *p* = 0.009) were significantly associated with poorer prognosis. 

Similarly, patients with elevated mGPS had a shorter overall survival (6.7, 12.9, and 20.7 months for mGPS 2, 1, and 0, respectively; *p* = 0.008).

The estimated median survival did not significantly differ between patient groups with high (≥2.6) and low (<2.6) LMR values (15.6 vs. 13.6 months, *p* = 0.053). 

**Figure 3:**
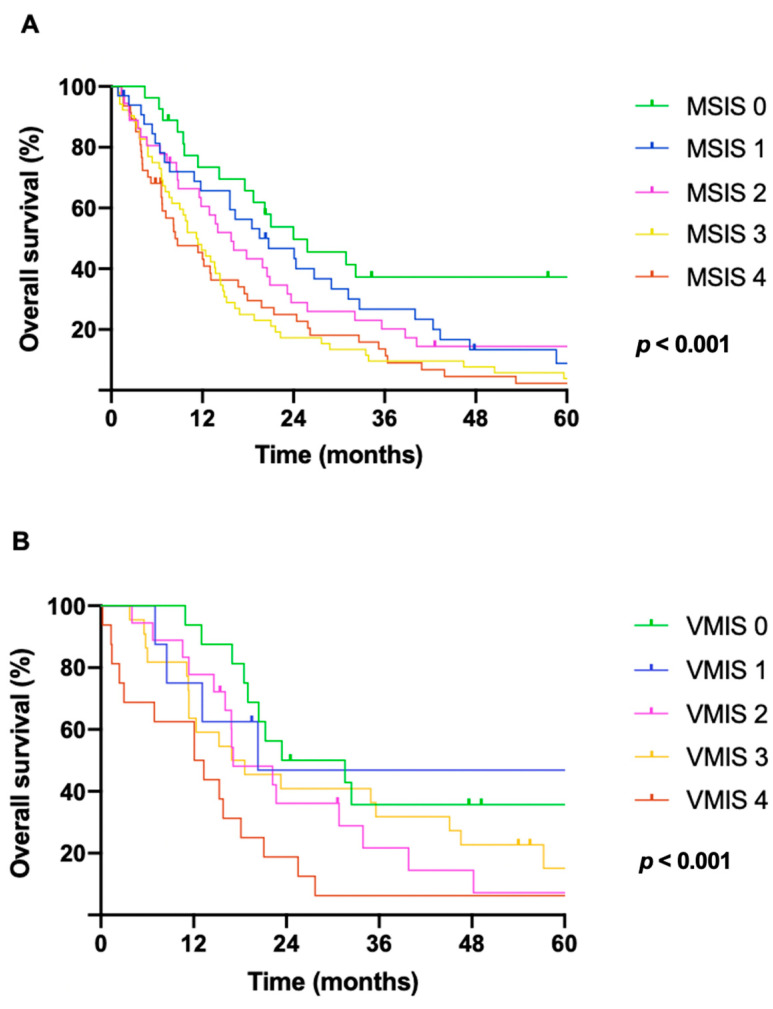
The Mesothelioma Systemic Inflammation Score is independently associated with overall survival (OS) of MPM patients. Kaplan–Meier survival analysis was performed for all patients grouped by the MSIS (including four variables: pretreatment NLR [cut-off value: 5], PLR [cut-off value: 160], fibrinogen [cut-off value: 537.5 mg/dL], and CRP [cut-off value: 1 mg/dL]). (**A**) Median OS was significantly decreased with higher scores (24.0 months [95% CI: 11.4–36.5], 20.7 months [95% CI: 10.3–31.1], 15.8 months [95% CI: 10.1–21.4], 11.2 months [95% CI: 8.1–14.3], and 8.4 months [95% CI: 3.2–13.6] for MSIS of 0, 1, 2, 3, and 4, respectively; *p* < 0.001). (**B**) Similarly, OS in the independent validation cohort significantly decreased with higher scores (23.4, 20.3, 17.2, 16.9, 12.1 months, for MSIS of 0, 1, 2, 3, and 4, respectively; *p* < 0.001). Abbreviations: MPM = malignant pleural mesothelioma; MSIS = Mesothelioma Systemic Inflammation Score; NLR = neutrophil-to-lymphocyte ratio; PLR = platelet-to-lymphocyte ratio; CRP = C-reactive protein.

MSIS was able to stratify patients with significant survival differences. As displayed in Figure 3A, the median OS was significantly decreased with higher scores (24.0 months, 95% CI: 11.4–36.5; 20.7 months, 95% CI: 10.3–31.1; 15.8 months, 95% CI: 10.1–21.4; 11.2 months, 95% CI: 8.1– 14.3; and 8.4 months, 95% CI: 3.2–13.6 for MSIS of 0, 1, 2, 3, and 4, respectively; *p* < 0.001).

The prognostic power of MSIS to predict survival differences was further validated in an independent cohort (*n* = 80). The results of the baseline analysis could be verified in the external validation cohort. The median OS in the validation cohort significantly decreased with higher scores (23.4, 20.3, 17.2, 16.9, and 12.1 months for MSIS of 0, 1, 2, 3, and 4, respectively; *p* < 0.001; Figure 3B). 

Furthermore, survival rates for patients with the lowest (0) and highest (4) MSIS were 74% and 46% at 1 year, 37% and 14% at 3 years, and 37% and 2% at 5 years, respectively. Higher pretreatment MSIS was associated with more advanced disease stages (*p* = 0.001). 

Clinical characteristics according to the Mesothelioma Systemic Inflammation Scores are shown in Table 3, whereby significant differences between MSIS groups were found regarding stage (*p* = 0.001) and the type of treatment (*p* = 0.047). Detailed OS of patients according to the MSIS in different treatment groups is listed in Table 4.

Potential prognostic factors were investigated by univariable analyses. Hereby, the following parameters were predictors for OS: age (*p* = 0.019), ECOG performance status (*p* < 0.001), histologic subtype (*p* = 0.006), tumor stage (*p* < 0.001), type of treatment (*p* < 0.001), mGPS (*p* = 0,008), NLR (*p* = 0.006), PLR (*p* = 0.009), fibrinogen (*p* < 0.001), CRP (*p* = 0.004), and MSIS (*p* < 0.001). As a next step, multivariable analysis was performed, wherein treatment (*p* = 0.004), stage (*p* = 0.001), and the MSIS (*p* < 0.001) were independently associated with OS (Table 5).

### 3.4. Pretreatment Msis Predicts Benefit of Surgery Within Multimodality Treatment Protocols

**Figure 4:**
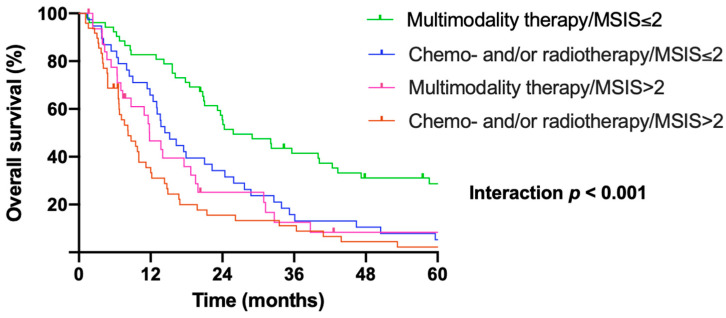
Low MSIS predicts survival benefit of surgery within multimodality treatment protocols. Kaplan–Meier survival analysis of subgroups of patients in regard to pretreatment MSIS (low ≤ 2 vs. elevated > 2) and treatment modalities (surgery within multimodality therapy vs. chemo- and/or radiotherapy). Patients with low MSIS had a significantly longer overall survival after surgery within multimodality treatment compared to patients after chemo- and/or radiotherapy alone (25.8 months, 95% CI: 16.4–35.3 vs. 14.4 months, 95% CI: 10.4–18.4; *p* < 0.001). In patients with elevated MSIS, the treatment strategies did not significantly influence the median overall survival (surgery within multimodality protocols: 11.8 months [95% CI: 8.3–15.3] vs. CTH and/or RTH: 8.2 months [95% CI: 5.2–11.3]; *p* = 0.233). Abbreviations: MSIS = Mesothelioma Systemic Inflammation Score; CHT = chemotherapy; RTH = radiotherapy.

To further validate the predictive value of the pretreatment MSIS in MPM, we performed a sub-analysis regarding treatment modalities and MSIS. Thus, patients were divided into four subgroups: (1) patients with low (≤2) MSIS undergoing surgery within multimodality treatment, (2) patients with low (≤2) MSIS undergoing chemo- and/or radiotherapy alone, (3) patients with elevated (>2) MSIS receiving surgery within multimodality treatment, and (4) patients with elevated (>2) MSIS receiving chemo- and/or radiotherapy alone. The pairwise comparison between the four groups revealed that significant survival benefit of aggressive multimodality regimens including surgery was limited to patients with low MSIS. The median survival was significantly different between these four groups, whereby patients with low MSIS receiving multimodality treatment including macroscopic complete resection showed the longest survival, while patients with high MSIS receiving CTH and/or RTH alone showed the shortest survival time (*p* < 0.001; Figure 4). Accordingly, patients with low MSIS had a significantly longer overall survival after undergoing surgery within multimodality treatment protocols compared to patients after chemo- and/or radiotherapy alone (25.8 months, 95% CI: 16.4–35.3 vs. 14.4 months, 95% CI: 10.4–18.4; *p* < 0.001). In contrast, the treatment strategies did not significantly influence the overall survival in patients with elevated MSIS (surgery within multimodality protocols: 11.8 months, 95% CI: 8.3–15.3 vs. CHT and/or RTH: 8.2 months, 95% CI: 5.2–11.3; *p* = 0.233). 

## 4. Discussion

In the present study, we investigated the prognostic and predictive power of a novel inflammation-based system—the Mesothelioma Systemic Inflammation Score, a product of NLR, PLR, CRP, and fibrinogen—in a large cohort of pleural mesothelioma patients treated in a high-volume center. Our results indicate that MSIS is an easily accessible, non-invasive, inexpensive, and reproducible tool in routine clinical management and is strongly associated with outcome prediction in MPM. 

Currently, there is a lack of accurate biomarkers and inflammatory scoring systems for diagnosis, patient selection, prognostication, and treatment prediction in mesothelioma [17,47,48].

Prognostication in MPM has been mainly studied in single-center settings and with a limited number of patients. Investigated factors have frequently included age, sex, histologic subtype, tumor stage, tumor site, performance status, and treatment approaches, as well as laboratory parameters and biomarkers such as white blood cell counts, platelet counts, CRP, fibrinogen, albumin, activin A, and combined inflammatory markers of those [9,17,18,19,20,49]. Male sex, non-epithelioid histology, high white blood cell and platelet counts, high CRP, low hemoglobin, chest pain, right-sided disease, and treatment without surgical resection have been validated as poor prognostic indicators across the literature [5,7,50,51,52]. Only a few advances in disease prognostication and prediction were reported in recent research, including the proposal of nuclear grading in epithelioid mesothelioma by the 2021 WHO Classification of Tumors of the Pleura [53]. Pathologic grading and morphological features such as necrosis and mitosis characteristics were proven as independent prognosticators with therapeutic implications for treatment allocation and clinical management [54,55,56].

In various malignancies, inflammation-based markers have been suggested as prognostic indicators of patients´ outcomes as inflammation plays a critical part in cancer progression. Neutrophil, lymphocyte, monocyte, and platelet counts as well as NLR, PLR, LMR, CRP, and the systemic immune-inflammation index (SII) have been verified as key inflammatory factors [57,58,59,60]. The association between NLR and outcome in MPM has been laboriously studied in smaller and larger-scale studies as well as in patient cohorts receiving systemic therapy or macroscopic complete resection [28,61,62,63,64]. The prognostic effect of NLR was further validated in a meta-analysis of a total of 11 studies involving 1533 patients [28]. The significance of high SII (calculated as neutrophil × platelet/lymphocyte counts) has been tested and correlated with unfavorable prognosis in MPM, as well as in non-small-cell lung cancer, esophageal squamous cell carcinoma, gastric cancer, and renal cell cancer [30,31,32,33,65]. 

In mesothelioma patients, high CRP and low albumin levels have been strongly associated with poor prognosis. The C-reactive protein/albumin ratio (CAR) has also been validated as an independent predictor [66,67]. Previously, our group verified elevated CRP as an independent marker of poor survival in MPM and identified the interaction between CRP and treatment modalities indicating a benefit of surgery within multimodality treatment in selected patients with normal pretreatment CRP levels. [25] Furthermore, the C-reactive protein/albumin ratio as well as the C-NR index (CRP × neutrophil counts) were correlated with histology and disease stage and were able to predict clinical outcome in patients who underwent extrapleural pneumonectomy as preoperative predictors [68]. 

Another clinically significant major prognostic score for patient stratification for multimodality treatment—the Multimodality Prognostic Score (MMPS)—also included CRP and albumin along with histology and tumor volume and was prospectively validated as a predictive score to allocate patients to surgery in pleural mesothelioma [69,70].

Formerly, we also investigated and found fibrinogen to be an independent prognostic biomarker in MPM with predictive value for treatment benefits achieved by macroscopic complete resection within multimodality therapy [18].

In spite of extensively ongoing research on biomarkers for diagnosis, prognosis, and clinical decisions in mesothelioma, there is no clear consensus in regard to their clinical implementation [71].

Moreover, pleural mesothelioma remains challenging to treat with poor prognosis despite recent developments in the field of immunotherapy and checkpoint inhibitors as well as less aggressive surgical techniques with reduced morbidity. A selected subgroup of patients (<10%) is eligible for radical multimodality treatment including macroscopic complete resection in combination with systemic chemo- and/or immunotherapy and/or radiotherapy with added survival benefit. 

The merits of multimodality treatment protocols have been endlessly discussed, often with limited evidence from prospective trials. The prospective Surgery for Mesothelioma After Radiation Therapy (SMART) trial was able to show that eligible patients can benefit from radical treatment modalities [72]. The highly anticipated results from the only randomized clinical trials evaluating the feasibility of surgery, the Mesothelioma and Radical Surgery (MARS) and MARS 2 trials, showed no benefit in survival time in patients who underwent surgery compared to patients treated medically [73,74]. However, several potential problems with the study have been highlighted since and it appears that the controversy about treatment decisions will continue. 

So far, treatment allocation in MPM has been subject to a number of unquantifiable parameters such as symptoms and performance status. Accurate biomarker prognostication could potentially improve clinical outcome by supporting early detection and treatment decisions offering multimodality therapy pathways or sparing patients from aggressive and potentially morbid treatment strategies [2,75,76,77,78,79]. 

A fair prognostic ability of NLR, PLR, CRP, and fibrinogen with respect to overall survival has been demonstrated in the literature in mesothelioma as well as in other malignancies. In our study, these predictors were also validated and found to be strongly associated with survival, thus being selected duly as a basis for a newly developed combined inflammation-based score that was assessed for its prognostic and predictive value in the present analysis in a large cohort in MPM. Moreover, the multivariable analysis determined that this novel inflammatory score was independently associated with survival. In agreement with evidence from the literature, we selected widely accepted, validated, predefined cut-off values to facilitate clinical applicability. The components of the MSIS are easily available and routinely measured in blood analyses in clinical work-up at the time of diagnosis before treatment decisions with the potential of identifying patients who may benefit from multimodality treatment protocols. MSIS in mesothelioma was able to indicate prognosis by stratifying patient groups with significant survival differences. Higher scores were significantly associated with reduced survival. Additionally, MSIS predicted a survival benefit of aggressive multimodality treatment including surgery in patients with low scores. Patients with low scores had a significantly longer overall survival when undergoing surgery within multimodality therapy compared to chemo- and/or radiotherapy alone, while both treatment subgroups with elevated scores had comparably poor survival. 

One limitation of this study is the retrospective single-center design; however, the score was developed based on a large number of patients with a constant treatment allocation algorithm. Treatment allocation was based on the recommendations of mesothelioma expert guidelines such as the National Comprehensive Cancer Network (NCCN) guidelines, the American Society of Clinical Oncology Clinical Practice Guidelines (ASCO), the ERS/ESTS/EACTS/ESTRO guidelines (European Respiratory Society [ERS]/European Society of Thoracic Surgeons [ESTS]/European Association for Cardio-Thoracic Surgery [EACTS]/European Society for Radiotherapy and Oncology [ESTRO]), and the ESMO Clinical Practice Guidelines (European Society for Medical Oncology). Due to the retrospective study design, treatment allocation may still reflect a selection bias. Validation of the score, ideally in a prospective setting, is warranted. Overall, these findings encourage the initiation of further large-scale investigations to prospectively assess the role of inflammation-based markers in terms of prediction in clinical practice and treatment decisions. Determination of serum markers should be a subject of extensive research as they are easy to obtain and may contribute to better patient selection in mesothelioma treatment. As described, existing evidence suggests that inflammation-related scoring systems are associated with high prognostic value, but limits still exist. The literature lacks prospective evaluations that are needed to confirm the results. Moreover, the ability of inflammation scoring to clarify the role of different treatment approaches in patients’ outcomes needs to be further investigated.

## 5. Conclusions

The Mesothelioma Systemic Inflammation Score predicted benefits from macroscopic complete resection as part of multimodality treatment in distinct patients.

## Figures and Tables

**Table 1:** Overview—the Mesothelioma Systemic Inflammation Score (MSIS).

MSIS (Score 0–4)	0 Point Each	1 Point Each
NLR	<5	≥5
PLR	<160	≥160
Fibrinogen (mg/dL)	<537.5	≥537.5
CRP (mg/dL)	<1	≥1

Abbreviations: NLR = neutrophil-to-lymphocyte ratio; PLR = platelet-to-lymphocyte ratio; CRP = C-reactive protein.

**Table 2:** Patient characteristics.

Demographics	*n* (%)
Age, y, median	67 (IQR 58–74)
Sex	
Male	147 (75)
Female	48 (25)
Histological subtype	
Epithelioid	144 (74)
Non-epithelioid	51 (26)
ECOG PS	
0	118 (60)
1	54 (28)
2	14 (7)
3	9 (5)
Stage	
Early (I–II) Late (III–IV)	96 (49) 99 (51)
Diagnostic method	
VATS Open thoracotomy	160 (82) 35 (18)
Treatment	
Surgery-based multimodality treatment	90 (46)
Chemo-and/or radiotherapy	80 (41)
Best supportive care	25 (13)
Symptoms	
Dyspnea Chest pain Cough	121 (62) 76 (39) 36 (19)
Pleural effusion	137 (70)
Site	
Left	106 (54)
Right	89 (46)

Abbreviations: IQR = interquartile range; ECOG PS = Eastern Cooperative Oncology Group performance status; VATS = video-assisted thoracoscopy.

**Table 3:** Patient characteristics and distribution according to the Mesothelioma Systemic Inflammation Scores.

	MSIS 0		MSIS 1		MSIS 2		MSIS 3		MSIS 4			Total	
Variables	*n*	%	*n*	%	*n*	%	*n*	%	*n*	%	*p*-Value	*n*%	
Age													
<65	16	59	14	42	16	44	21	40	18	38	0.484	85	44
>65	11	41	19	58	20	56	31	60	29	62		110	56
Sex													
Male	21	78	24	73	22	61	39	75	41	87	0.104	147	75
Female	6	22	9	27	14	39	13	25	6	13		48	25
ECOG PS													
0	20	74	17	52	24	67	32	61	23	50	0.174	116	59
1	6	22	12	36	8	22	16	31	18	38		60	31
2	1	4	3	9	3	8	3	6	3	6		13	7
3	0	0	1	3	1	3	1	2	3	6		6	3
Histology													
Epithelioid	25	93	23	70	28	78	36	69	32	68	0.145	144	74
Non-epithelioid	2	7	10	30	8	22	16	31	15	32		51	26
Stage													
I	16	59	19	58	13	36	18	35	15	32	0.001	81	41
II	4	15	3	9	3	8	2	4	3	6		15	8
III	7	26	11	33	19	53	28	54	21	45		86	44
IV	0	0	0	0	1	3	4	7	8	17		13	7
Treatment													
Surgery-based MMT	19	70	17	52	16	44	25	48	13	28	0.047	90	46
CTH and/or RTH	4	15	13	39	15	42	21	40	27	59		80	41
BSC	4	15	3	9	5	14	6	12	6	13		25	13

Abbreviations: ECOG PS = European Cooperative Oncology Group performance status; MMT = multimodality treatment; CTH = chemotherapy; RTH = radiotherapy; BSC = best supportive care. Kruskal–Wallis test was used to compare the influencing factors.

**Table 4:** Detailed median overall survival according to the Mesothelioma Systemic Inflammation Scores in the different treatment groups.

	Patients Treated with Surgery Within Multimodality Protocols	Patients Treated with Chemo- and/or Radiotherapy	Patients Received Best Supportive Care
	*n* = 90	*n* = 80	*n* = 25
Scores	Median OS in months (95% CI)		
MSIS 0	73.4 (0.0–163.4)	18.7 (16.9–20.6)	16.3 (0.0–41.1)
MSIS 1	29.0 (7.6–50.4)	7.0 (0.0–14.8)	9.6 (7.7–11.5)
MSIS 2	23.2 (17.7–28.7)	11.8 (6.4–17.2)	8.8 (0.0–20.2)
MSIS 3	14.4 (11.9–16.9)	7.6 (3.6–11.7)	6.6 (0.0–15.8)
MSIS 4	11.5 (8.9–14.2)	6.7 (2.0–12.4)	3.2 (1.4–5.1)

Abbreviations: MSIS = Mesothelioma Systemic Inflammation Score; OS: overall survival; CI = confidence interval.

**Table 5:** Univariable and multivariable analyses of prognostic factors for overall survival.

Variables			Univariable			Multivariable
	*n*	OS (95% CI)	*p*-Value	HR	95% CI	*p*-Value
Age			0.019	1.4	1.1–1.9	0.780
>65	110	11.8 (8.0–15.6)				
<65	85	17.9 (12.5–23.3)				
Sex			0.078	0.7	0.5–1.0	
Female	48	15.6 (12.4–18.8)				
Male	147	13.6 (10.7–16.5)				
ECOG PS			<0.001	1.5	1.2–1.9	0.129
0	116	16.1 (12.7–19.5)				
1	60	14.0 (9.6–18.5)				
2	13	8.8 (2.8–14.9)				
3	6	1.4 (0.5–2.2)				
Histology			0.006	0.6	0.4–0.9	0.141
Epithelioid	144	14.3 (10.5–18.1)				
Non-epithelioid	51	12.2 (6.1–18.3)				
Stage			<0.001	1.6	1.3–1.8	0.001
I	81	21.4 (15.2–27.6)				
II	15	19.9 (13.0–26.8)				
III	86	11.4 (9.2–13.6)				
IV	13	3.0 (1.3–4.8)				
Treatment			<0.001	1.9	1.5–2.3	0.004
Surgery-based MMT	90	22.3 (18.6–26.0)				
CTH and/or RTH	80	9.8 (6.7–12.8)				
BSC	25	8.8 (4.2–13.5)				
mGPS			0.008	1.3	1.1–1.6	0.945
0	62	20.7 (15.4–26.1)				
1	92	12.9 (11.2–14.6)				
2	41	6.7 (2.6–10.8)				
NLR			0.006	1.5	1.1–2.1	0.272
High (≥5)	70	11.4 (6.7–16.1)				
Low (<5)	125	16.1 (12.4–19.9)				
PLR			0.009	1.6	1.1–2.2	0.139
High (≥160)	53	12.9 (10.9–14.9)				
Low (<160)	142	20.2 (14.3–26.1)				
LMR			0.053	0.7	0.5–1.0	
High (≥2.6)	66	15.6 (10.2–21.0)				
Low (<2.6)	129	13.6 (10.8–16.4)				
Fibrinogen			<0.001	1.7	1.3–2.4	0.125
High (≥537.5 mg/dL)	107	10.0 (7.2–12.8)				
Low (<537.5 mg/dL)	88	20.5 (13.8–27.3)				
CRP			0.004	1.6	1.2–2.2	0.164
High (≥1 mg/dL)	134	11.9 (9.4–14.3)				
Low (<1 mg/dL)	61	20.5 (15.7–25.3)				
MSIS			<0.001	1.2	1.1–1.4	<0.001
0	27	24.0 (11.4–36.5)				
1	33	20.7 (10.3–31.1)				
2	36	15.8 (10.1–21.4)				
3 4	52 47	11.2 (8.1–14.3) 8.4 (3.2–13.6)				

Abbreviations: OS = overall survival; CI = confidence interval; HR = hazard ratio; ECOG PS = European Cooperative Oncology Group performance status; MMT = multimodality treatment; CTH = chemotherapy; RTH = radiotherapy; BSC = best supportive care; mGPS = modified Glasgow prognostic score; NLR = neutrophil-to-lymphocyte ratio; PLR = platelet-to-lymphocyte ratio; LMR = lymphocyte-to-monocyte ratio; CRP = C-reactive protein; MSIS = Mesothelioma Systemic Inflammation Score (consisting of NLR, PLR, fibrinogen, and CRP).

## Data Availability

The data that support the findings of this study are available on request from the corresponding author (B.M.) upon reasonable request. The data are not publicly available due to privacy of the research participants.
